# *Vibrio vulnificus* infection complicated by acute-on-chronic liver failure: A case report

**DOI:** 10.1097/MD.0000000000039980

**Published:** 2024-10-04

**Authors:** Tingting Wang, Qifeng Huang

**Affiliations:** a Department of Nephrology, The First Affiliated Hospital of Hainan Medical University, Haikou, China; b Department of Emergency, The First Affiliated Hospital of Hainan Medical University, Haikou, China.

**Keywords:** acute liver failure, case report, necrotizing soft tissue infection, *Vibrio Vulnificus*

## Abstract

**Rationale:**

*Vibrio vulnificus* is a gram-negative bacterium that can cause 3 clinical syndromes: gastrointestinal symptoms, skin septicemia, and primary septicemia. *V vulnificus* infection can induce an exacerbation of liver disease, eventually requiring intensive care for multiorgan failure.

**Patient concerns:**

A 56-year-old Chinese male who was admitted for left lower limb swelling 5 days after sustaining an injury. His left lower leg was wounded with a machete used for cutting rubber. Notably, this machete had also been previously utilized for cutting seafood. Blood culture results indicated the presence of *V vulnificus* during the hospitalization. The patient’s condition deteriorated rapidly leading to acute liver failure. Over the ensuing days, the patient experienced separation of tendency of aminotransferase and bilirubin (bilirubin-aminotransferase dissociation), indicative of worsening liver function. Of note, the patient had a history of untreated hepatitis B virus infection and a long drinking history.

**Diagnoses:**

Acute-on-chronic liver failure following a *V vulnificus* infection.

**Interventions:**

We utilized double plasma molecular adsorption system (DPMAS) to address the deterioration of the patient’s liver function.

**Outcomes:**

After 2 DPMAS treatments, the patient’s liver function showed improvement.

**Lessons:**

This report underscores the importance of timely and repeated DPMAS treatment of patients with a drinking history or chronic liver disease when they present with *V vulnificus* septicemia.

## 1. Introduction

*Vibrio vulnificus* is a Gram-negative bacterium typically known for inducing gastrointestinal symptoms, skin and soft tissue necrosis, and septicemia. Initially regarded as a foodborne pathogen, *V vulnificus* has emerged as an underrecognized zoonotic pathogen. Further research has shown that it not only affects marine animals but is also present in contaminated seawater or on surfaces that have been in contact with infected materials. It can enter the bloodstream through skin wounds, leading to human infection.^[1]^ The clinical manifestations of *V vulnificus* infection typically include nausea, vomiting, gastrointestinal cramps, diarrhea, fever, and chills. Traumatic infections may present with skin swelling, pain, deep muscle infection, and peripheral tissue necrosis, and in severe cases, involvement of other organs may occur.^[2]^ The mortality rate of sepsis caused by *V vulnificus* exceeds 50%, while that of wound infections is above 15%.^[3]^ Clinical management includes wound management and systemic antibiotic therapy.^[4,5]^ Notably, in patients with underlying diseases such as liver disease, diabetes, end-stage renal disease, cirrhosis, and immunological disorders, *V vulnificus* infection can induce an exacerbation of these comorbidities. *Vibrio vulnificus* possesses multiple virulence factors, including the production of an anti-phagocytic polysaccharide capsule, *V vulnificus* proteolytic enzyme (VVPE), *V vulnificus* hemolysin A (VVHA), and the multifunctional autoprocessing repeats-in-toxins (MARTX) toxin, and systems for iron availability and iron acquisition.^[6]^ Of note, these virulence factors are associated with inducing liver damage in humans. Herein, we present a rare case of a male with chronic Hepatitis B virus infection who developed liver failure subsequent to *V vulnificus* infection, culminating in multi-organ failure necessitating intensive care.

## 2. Case report

**Figure 1.**
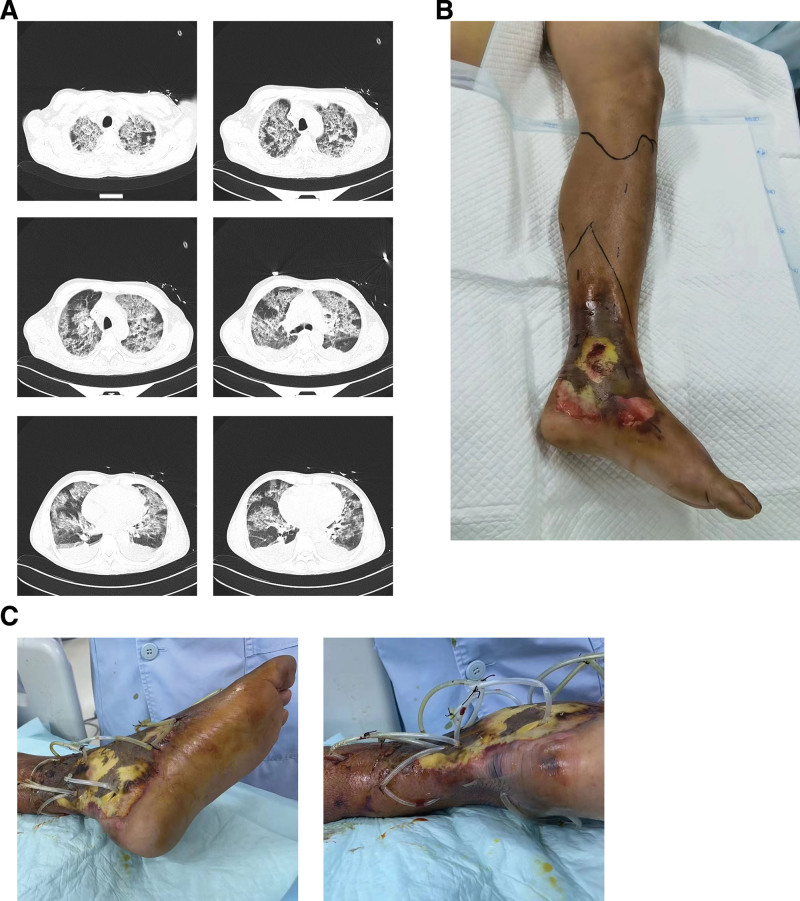
(A) Chest computed tomography (CT) on the 7th day of the patient. (B) Skin infection and tissue necrosis of the left lower. (C) Incision and drainage surgery for infection of the left.

A 56-year-old Chinese male, employed as a rubber plantation worker, presented to our emergency department with swelling in his left lower limb, 5 days after sustaining an injury. One week prior, he had accidentally wounded his left lower leg with a machete used for cutting rubber. Notably, this machete had also been previously utilized for cutting seafood. Although the wound had initially healed, he began experiencing swelling and pain in his left lower limb, accompanied by increased local skin temperature, and systemic symptoms, including chills, fever, and limb weakness 5 days prior to admission. The patient has a history of hepatitis B virus infection that was not treated with antiviral medication. Moreover, he has a smoking history of over 30 years, averaging about 30 cigarettes per day, and a drinking history exceeding 30 years, with an average daily consumption of approximately 70 mL of alcohol. Upon admission, the patient was alert and coherent, with a respiratory rate of 20 breaths per minute, a pulse rate of 110 bpm, a blood pressure of 134/76 mm Hg, and an oxygen saturation of 97%. Coarse breath sounds and occasional wet rales were noted upon lung auscultation. Abdominal examination revealed no abnormalities. The left lower limb exhibited swelling, increased local skin temperature, tenderness, and exudation, but no signs of active bleeding were observed. Initial chest computed tomography (CT) scans revealed multiple pulmonary infiltrates and areas of consolidation, notably prominent in the upper lobes of both lungs (Fig. 1A). Empirical anti-infective therapy (piperacillin-tazobactam) was initiated following the collection of purulent secretions from the left lower limb and blood samples from the extremities to be cultured. However, over the subsequent 72 hours, the patient’s clinical status deteriorated, characterized by the onset of black tarry stools and hypotension necessitating vasopressor support. Subsequently, he was transferred to the intensive care unit. On the fourth day, manual blood culture method results indicated the presence of *V vulnificus*. Consequently, The antibiotic regimen was altered from piperacillin-tazobactam, administered at 4.5 g every 8 hours, to a combination therapy consisting of imipenem-cilastatin at 2.0 g every 6 hours, moxifloxacin at 4.0 g daily, vancomycin at 500 mg every 8 hours, and fluconazole at 0.4 g daily, aimed at targeted anti-infective treatment. Due to skin and tissue necrosis of the left lower limb (Fig. 1B), bedside incision and drainage were performed, complemented by continuous negative pressure suction (Fig. 1C). Continuous blood purification therapy was administered using a cytosorbents apheresis 330 (CA330) cytokine adsorber to mitigate cytokine-mediated complications. On the fifth day, the patient exhibited pronounced dyspnea, accompanied by thick and difficult-to-expel sputum, necessitating tracheal intubation and ventilator support. Concurrently, elevations in alanine aminotransferase (ALT) and aspartate aminotransferase (AST) levels, total bilirubin (TBil) and direct bilirubin (DBil), and abnormalities in coagulation function were noted. By the seventh day, while ALT and AST levels exhibited a downward trend, total and direct bilirubin levels continued to rise. Serum hyaluronic acid, type III procollagen N-terminal peptide, type IV collagen, and laminin were all elevated, but a liver CT did not indicate cirrhosis. After ruling out other possible causes of liver damage, we calculated the Child-Pugh score, which was 9 points, and the chronic liver failure patients sequential organ failure assessment (CLIF-SOFA), which was 16 points. Subsequently, double plasma molecular adsorption system (DPMAS) was swiftly initiated. By the eighth day, a notable decline in total bilirubin levels was observed, prompting discontinuation of DPMAS. However, on the 11th day, given the persistent need for liver support, DPMAS was reinstated, yielding some improvement in liver function. On the 14th day, the family requested to discontinue all treatments and discharge the patient due to the severity of the patient’s condition and financial constraints. The patient died at home 4 hours after discharge.

## 3. Discussion

*Vibrio vulnificus* infection is associated with a high mortality rate and presents with a range of clinical manifestations, including gastrointestinal symptoms, cutaneous sepsis, and primary sepsis. Gastrointestinal symptoms are typically characterized by diarrhea, nausea, and vomiting, while systemic manifestations often include fever and chills. Notably, over 50% of affected individuals develop severe cellulitis, which is frequently accompanied by ecchymosis and hemorrhagic bulla formation. Hospitalization becomes imperative when septic shock ensues.^[7]^

This case presents an instance of septic shock and acute liver failure resulting from an infection induced by *V vulnificus* following a well-documented history of trauma. The infection initially began with a minor wound that seemingly healed before progressing to a skin and tissue infection. Overall, this case underscores the lethality of *V vulnificus* infections, as the onset of symptoms often heralds a state of sepsis or septic shock, posing a significant risk of severe multi-organ dysfunction.

**Figure 2.**
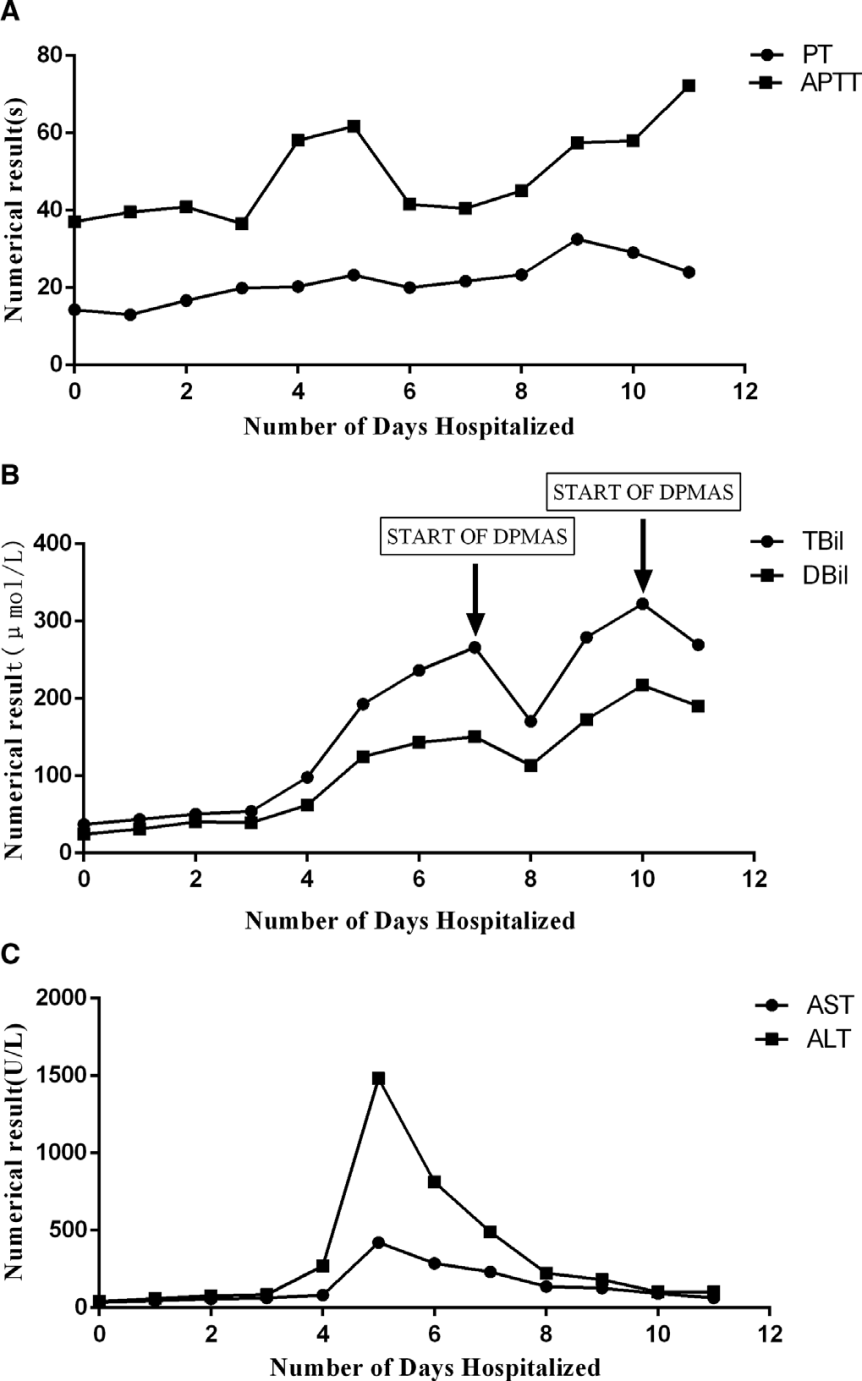
(A) The trends of PT and APTT over time. (B) The trends of TBil and DBil over time. Both parameters showed a decrease on the 7th and 10th days after DPMAS. (C) The trends of AST and ALT over time. The highest values were observed on the 5th day of hospitalization, followed by a decline. ALT = alanine aminotransferase, APTT = activated partial thromboplastin time, AST = aspartate aminotransferase, DBil = direct bilirubin, DPMAS = double plasma molecular adsorption system, PT = prothrombin time, TBil = total bilirubin.

The diagnosis of *V vulnificus* is confirmed through cultures of blood, stool, and secretions.^[8]^ By the time the pathogen was identified, the patient had already developed severe sepsis, accompanied by complications such as gastrointestinal bleeding, respiratory failure, and liver failure. Throughout the hospitalization, daily coagulation function (Fig. 2A), bilirubin (Fig. 2B), and transaminases (Fig. 2C) tests were performed. During hospitalization, prothrombin time (PT) and activated partial thromboplastin time (APTT) levels were significantly elevated (Fig. 2A), indicating an increased risk of bleeding. TBil and DBil showed an upward trend during hospitalization. We administered DPMAS treatment on the 7th and 10th days, respectively. The levels of these 2 parameters decreased the day after treatment but continued to increase thereafter (Fig. 2B). AST and ALT reached their peak values on the 5th day of hospitalization, then declined (Fig. 2C). After receiving DPMAS treatment, the patient’s liver function showed improvement. The term “bilirubin-aminotransferase dissociation” refers to the phenomenon commonly observed during the progression of hepatitis, where extensive necrosis of liver cells leads to a progressive decline in the liver’s ability to process bilirubin, resulting in an increase in bilirubin levels. In assessing potential causes of liver failure, our initial attention was directed towards the patient’s chronic hepatitis, which had not been systematically treated with antivirals.

Some studies indicate that mild infections can become severe in immunosuppressed patients, particularly those with chronic hepatitis, due to high transferrin saturation in their blood, which aids microbial invasion of multiple organs.^[9]^ Emerging evidence suggests that nutritional immunity mediated by ferritin, which chelates iron, may play a pivotal role in combating *V vulnificus* infection. During chronic hepatitis or cirrhosis, the liver’s protein synthesis capabilities are reduced, leading to an overload of serum iron ions, further facilitating *V vulnificus* septicemia.^[10]^ The concentration of iron in the internal environment can influence the growth and migration of VVPE, an extracellular protease of *V vulnificus*, can degrade various proteins such as mucin and hemoglobin, enhancing the adherence of *V vulnificus* in tissues.^[11,12]^ This suggests that in patients with chronic liver disease or cirrhosis, a decrease in liver physiological functions could unbalance the already fragile compensatory mechanisms during a *V vulnificus* infection, leading to acute-on-chronic liver failure.

However, in our case, the patient initially presented to the emergency department with normal liver function, which later deteriorated as the condition progressed. Although liver function improved following DPMAS treatment, the emergence of separation of tendency of aminotransferase and bilirubin was indicative of liver failure. This highlights the importance of timely and repeated DPMAS treatments, suggesting that early intervention might reverse disease progression. Despite the unsuccessful outcome, our case offers significant insights for clinicians. It underscores the importance of promptly conducting etiological testing and administering suitable antibiotic and life support treatments to patients with a history of alcohol consumption or liver disease when they present with *V vulnificus* septicemia.

## Author contributions

**Conceptualization:** Tingting Wang.

**Investigation:** Qifeng Huang.

**Writing – original draft:** Tingting Wang.

**Writing – review & editing:** Qifeng Huang.
